# Informing Integration of Genomic Medicine Into Primary Care: An Assessment of Current Practice, Attitudes, and Desired Resources

**DOI:** 10.3389/fgene.2019.01189

**Published:** 2019-11-21

**Authors:** June C. Carroll, Judith Allanson, Shawna Morrison, Fiona A. Miller, Brenda J. Wilson, Joanne A. Permaul, Deanna Telner

**Affiliations:** ^1^Sinai Health System, Department of Family and Community Medicine, University of Toronto, Toronto, ON, Canada; ^2^Department of Genetics, Children’s Hospital of Eastern Ontario, Ottawa, ON, Canada; ^3^Institute of Health Policy, Management and Evaluation, University of Toronto, Toronto, ON, Canada; ^4^Division of Community Health and Humanities, Memorial University of Newfoundland, St. John’s, NL, Canada; ^5^Sinai Health System, Ray D Wolfe Department of Family Medicine, Toronto, ON, Canada; ^6^South East Toronto Family Health Team, Department of Family and Community Medicine, University of Toronto, Toronto, ON, Canada

**Keywords:** primary health care, genomics, genetic services, health services needs, questionnaire

## Abstract

**Introduction:** Preparing primary care providers for genomic medicine (GM) first requires assessment of their educational needs in order to provide clear, purposeful direction and justify educational activities. More understanding is needed about primary care providers’ perspectives on their role in newer areas of GM and what resources would be helpful in practice. Our objective was to determine family physicians’ (FP) current involvement and confidence in GM, attitudes regarding its clinical value, suggestions for integration of GM into practice, and resources and education required.

**Methods**: A self-complete anonymous questionnaire was mailed to a random sample of 2,000 FPs in Ontario, Canada in September 2012.

**Results:** Adjusted response rate was 26% (361/1,365), mean age was 51, and 53% were male. FPs reported many aspects of traditional GM as part of current practice (eliciting family history: 93%; deciding who to refer to genetics: 94%; but few reported confidence (44%, 32% respectively). Newer areas of GM were not part of most FPs’ current practice and confidence was low (pharmacogenetics: 28% part of practice, 5% confident; direct-to-consumer genetic testing: 14%/2%; whole genome sequencing: 8%/2%). Attitudes were mixed with 59% agreeing that GM would improve patient health outcomes, 41% seeing benefits to genetic testing, but only 36% agreeing it was their responsibility to incorporate GM into practice. Few could identify useful sources of genetic information (22%) or find information about genetic tests (21%). Educational resources participants anticipated would be useful included contact information for local genetics clinics (89%), summaries of genetic disorders (86%), and genetic referral (85%) and testing (86%) criteria. About 58% were interested in learning about new genetic technologies. Most (76%) wanted to learn through in-person teaching (lectures, seminars etc.), 66% wanted contact with a local genetic counselor to answer questions, and 59% were interested in a genetics education website.

**Conclusion**: FPs lack confidence in GM skills needed for practice, particularly in emerging areas of GM. They see their role as making appropriate referrals, are somewhat optimistic about the contribution GM may make to patient care, but express caution about its current clinical benefits. There is a need for evidence-based educational resources integrated into primary care and improved communication with genetic specialists.

## Introduction

Genomic medicine (GM) is anticipated to profoundly affect medical practice. Primary care providers (PCPs), as first contact with the health care system and key to continuous and coordinated care, will be critical to the effective and appropriate implementation of GM. In studies over a decade ago, PCPs described how they would play an increasing role in GM. Essential skills identified by PCPs at that time included taking a family history, assessing genetic risk, providing a gatekeeping function by deciding who is appropriate for referral to genetics, providing patient support and coordinating surveillance and management.(Emery et al., 1999; Carroll et al., 2003) Over the subsequent years, integration of GM into clinical practice, including primary care, has been slow. A key reason for this is the lack of evidence of clinical utility of many genetic tests, but barriers and challenges to primary care implementation also include concern about the ethical, legal, and social implications of genetic testing, lack of PCP knowledge and skills, systems issues (e.g. time), and lack of awareness of genetic services. (Delikurt et al., 2015; Mikat-Stevens et al., 2015) PCPs and genetics experts acknowledge that PCPs need more knowledge in the area of genomics.(Emery et al., 1999; Carroll et al., 2003; Skirton et al., 2010; Houwink et al., 2011; Carroll et al., 2016a). Recognizing that a disease might be hereditary, indications for genetics referral and benefits and limitations of genetic tests ranked highest in a study of educational needs for general practitioners by a heterogeneous panel of experts.(Houwink et al., 2012) Core competencies in GM for health professionals have been developed.(Skirton et al., 2010; Korf et al., 2014) There is agreement that strategies to enable the appropriate integration of GM into primary care require more than merely addressing a knowledge deficit, but must also address attitudes and propose new systems of care to facilitate practice. These proposed “roadmaps” include training and education but also innovative systemic changes such as integration of genomic results into the electronic health record (EHR) with clinical decision support, and new models of delivering genetic services such as genetic counselors or nurses embedded in primary care clinics or made available through telephone helplines, etc. (Battista et al., 2012; Manolio et al., 2013; Houwink et al., 2013; David et al., 2015)

Preparing PCPs for GM first requires an assessment of their educational needs, in order to provide clear and purposeful direction and to justify educational activities. Little is known about what role PCPs see for themselves in the rapidly changing landscape of GM including pharmacogenomics, direct-to-consumer genetic testing and whole genome sequencing, or what system changes they think might be helpful and would be willing to incorporate in their practices. Our objectives were to determine family physicians’ (FP) current involvement in GM, confidence in GM primary care competencies, attitudes regarding the clinical importance of GM, awareness of genetic services, resources required, and suggestions for changes that would enable integration of GM into practice.

## Materials and Methods

### Questionnaire Design and Administration

This study used a self-complete, anonymous questionnaire which was developed by a multidisciplinary team. Where possible, questions were derived from the literature or previous questionnaires. (Carroll et al., 2009; Carroll et al., 2011) The questionnaire was divided into eight sections: current role and confidence in the tasks of each role providing genetic services in their practices (14 questions), completion of family history (2 questions), attitudes toward GM (11 questions), awareness of and experience with genetic services (12 questions), knowledge (18 questions), education and resources required (37 questions), and demographics (18 questions). Answers were a mixture of 3–5 point Likert scales (confidence, attitudes, awareness, resources), yes/no (experience), and multiple choice (knowledge). The knowledge component of the questionnaire consisted of 10 clinical vignettes with an accompanying question (4 cancer; 1 inheritance; 2 prenatal; 1 pediatric; 1 consanguinity; 1 adult onset disorder). One question asked “What would help you integrate genomic medicine into your practice in the future?” Several options were listed that were derived from the literature (Battista et al., 2012) as well as the research team, with a box to add “other” suggestions. Questions were pilot tested for face and content validity with 20 FPs from three practices.

In the body of the questionnaire we defined genomics as “the study of genes, their function and their interaction with all the other genes in the genome and the environment.” GM was defined as medicine that “uses genomic information and technologies (e.g. DNA sequencing) to determine an individual’s risk, predisposition, diagnosis and prognosis, and the selection and prioritization of therapeutic options (e.g. pharmacogenetic testing prior to administration of certain medications).”

The study was conducted from September 2012 to April 2013. Questionnaires were mailed to a random sample of 2,000 Ontario FPs taken from Scott’s Directory of Canadian physicians. A modified Dillman Method was employed (Dillman et al., 2009) including an introductory letter, questionnaire package 1 week later with instructions for a web link if preferred for questionnaire completion, a postcard reminder/thank you 2 weeks following the questionnaire, a second questionnaire package to non-responders 4 weeks following the postcard, and final mailed reminder 8 weeks later. As a token of appreciation, once a completed questionnaire was received, the respondent was entered into a draw to win one of twenty $150 Amazon Canada gift cards. FPs were considered eligible if they were in active full-time or part-time practice of family medicine in Ontario, Canada. Ethics approval was obtained from the Children’s Hospital of Eastern Ontario Research Ethics Board.

### Statistical Analysis

Completed questionnaires were coded, data were entered into an Excel spreadsheet, and analyzed using IBM SPSS, version 23 (IBM Corporation, Armonk, New York, USA, 2015).

Five-point Likert scales were collapsed into binary data by combining levels 4 and 5 for confidence variables as “confident” in skills, for attitudes and awareness variables as “agree/strongly agree,” for interest in education variables as “moderate/high,” for genetics resources as “useful/very useful.” A confidence score was created from items 1–10 of **Table 2**. These items were chosen as they were considered current core GM skills. We did not include newer skills related to pharmacogenomics and direct-to-consumer testing. One point was given for a rating of 4 or 5 on a confidence item, with a total score of ≥5/10 items indicating a “high” confidence score. A knowledge score that was greater than 7/10 correct was categorized as “high.”

Frequency distributions provided a descriptive analysis of the data. Correlation analysis was used to establish if there was an association between high knowledge and high confidence. Chi-squared analyses were conducted to look for associations between demographic variables and outcomes. Variables with significant associations were entered into binary logistic regression models to determine if they were predictors of confidence, attitudes, awareness, knowledge, and education and resources regarding GM. Covariates included in the model were older age (≤50/ > 50 years), younger age (≤40/> 40 years), sex (male/female), years in practice (<15/≥15 years), practice location (urban – population ≥ 500K/rural – population < 500K), practice type (solo/group or other), focused practice (yes/no), involved in teaching (yes/no), use electronic medical record (EMR) (yes/no), formal education in genetics (yes/no), continuing medical education in genetics in the last 5 years (yes/no), special interest in genetics (yes/no), and genetic condition in a close family member (yes/no).

## Results

### Demographics

**Figure 1:**
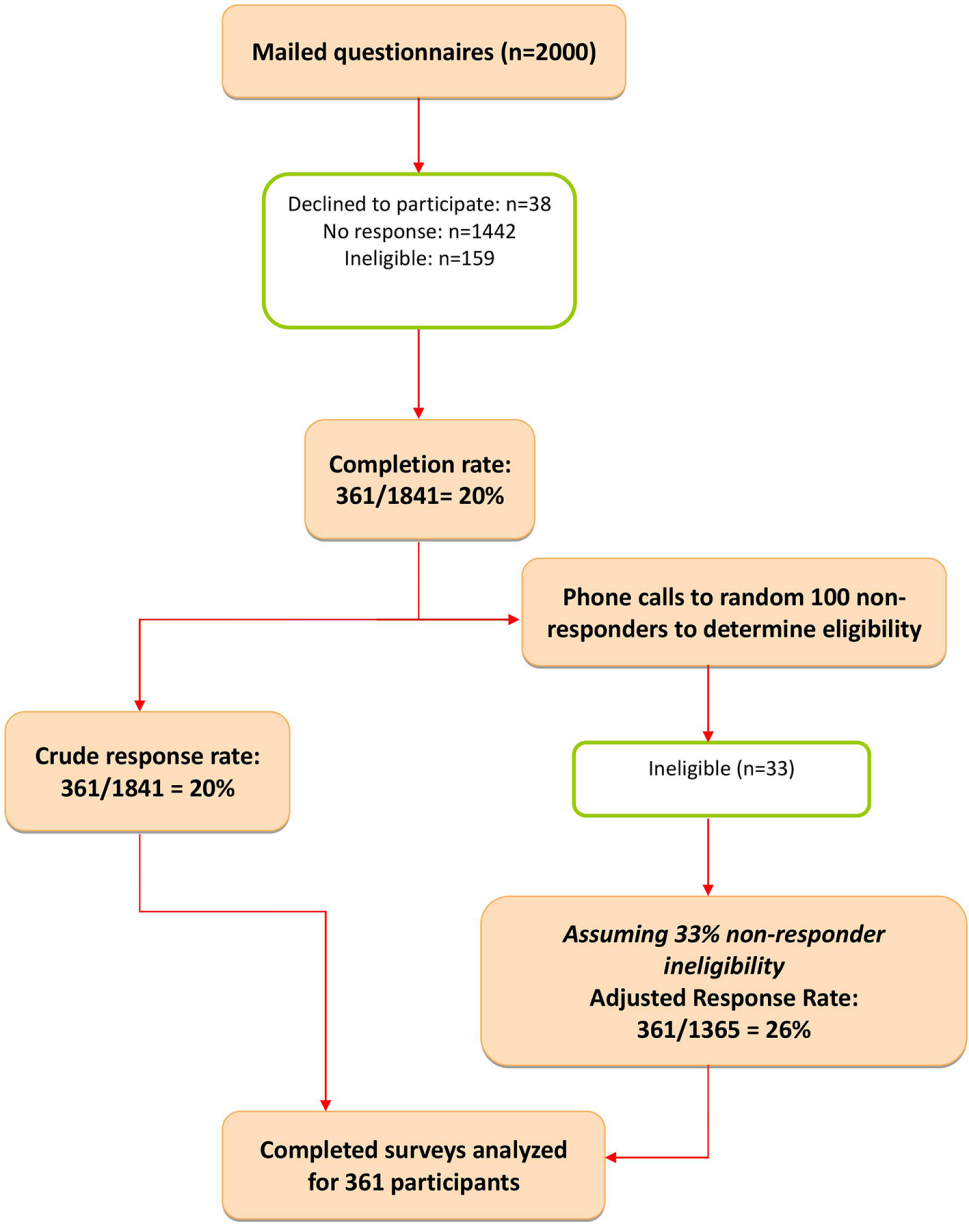
Response rate flowchart.

In total, 2,000 surveys were mailed, of which 159 were ineligible: wrong address, not in active practice or deceased, not practicing in Ontario, or belonged to excluded specialties. Of the remaining 1841 questionnaires, 361 were returned completed, giving a raw response rate of 19.6%. A random sample of 100 of the 1,442 non-responders was contacted by the project manager (SM) to determine if they met the eligibility criteria. Of those, 33 of the 100 contacted were not eligible for the reasons listed above. We then assumed that approximately 33% of the total non-responder group would also be ineligible, giving an adjusted response rate of 26.4% (361/1,365 eligible FPs) (**Figure 1**).

Demographics of respondents are shown in **Table 1**. Mean age was 51 years, with 53.2% male. Most (72.5%) had no formal education in genetics, but a small proportion indicated a special interest in genetics (18.3%), presence of a genetic condition in a close family member (20.7%) or had personally seen a genetic counselor or geneticist (10.6%).

**Table 1:** Participant demographics (n = 361)*.

Characteristic	Mean (SD)	Range
Age	50.9 (11.72)	Range: 27–77 yrs
	**N**	**%**
Sex: male	185/348	53.2
Size of practice community ≥500,000	157/351	44.7
Type of practice: solo	81/350	23.1
Focused practice >50%	54/338	16.0
Involved in teaching	192/353	54.4
Some formal education in genetics	94/342	27.5
Continuing education in genetics in last 5 yrs	57/352	16.2
Special interest in genetics	64/349	18.3
Genetic condition in a close family member	72/348	20.7
Personally seen a genetic counsel or/geneticist for concern related to personal or family health history	37/350	10.6

*Includes all respondents including family physicians (FPs) in focused practice.

### Current Role in Genomic Medicine

Participating FPs reported high involvement in some aspects of traditional GM (eliciting FH (93.3%)), identifying individuals with genetic conditions (89.5%), deciding who should be offered genetic referral (93.8%), knowing where to refer for genetic counseling (91.9%), and providing support to a patient coping with a genetic test result (82.8%) (**Table 2**). Most respondents (69.2%) reported completing a family history on 100% of new patients, with 72.6% reporting they routinely updated the family history yearly or at the periodic health exam.

**Table 2:** Current role in delivering genomic medicine and confidence with each task*.

Role	Part of current practice (yes)		Level of confidence with task (high = 4 or 5 on Likert scale)	
	N	%	N	%
1. Eliciting information about genetic conditions as part of a family or medical history	263/282	93.3	122/277	44.0
2. Identifying individuals with a genetic condition	246/275	89.5	59/277	21.3
3. Deciding who should be offered referral for genetic counseling or testing based on personal or family health history	256/273	93.8	89/278	32.0
4. Knowing where to refer for genetic counseling/genetic assessment	249/271	91.9	151/273	55.3
5. Providing support to patients coping with a genetic test result	227/274	82.8	82/273	30.0
6. Evaluating the clinical usefulness of a genetic test	144/271	53.1	40/256	15.6
7. Discussing the benefits, risks, and limitations of genetic testing with patients	180/273	65.9	43/265	16.2
8. Describing what to expect at a genetic counseling session	169/273	61.9	57/265	21.5
9. Obtaining credible, current information about genetics	134/259	51.7	25/235	10.6
10. Providing education about genetic conditions to patients	184/272	67.6	45/265	17.0
11. Discussing genetic variation in drug response with patients (e.g. pharmacogenetics)	74/264	28.0	10/224	4.5
12. Discussing the risks, benefits and limitations of “Direct-to-Consumer” genomic testing with patients	44/263	16.7	7/213	3.3
13. Discussing the interpretation of “Direct-to-Consumer” genomic test results with patients	37/263	14.1	4/212	1.9
14. Discussing the interpretation of whole genome sequencing with patients	20/262	7.6	4/208	1.9

*Includes only respondents who indicated they were not in focused practice, i.e. provided full scope family medicine.

However, reported involvement in some GM tasks was more limited with fewer than two-thirds saying that evaluating the use of a genetic test, discussing benefits, risks, and limitations of genetic testing with patients, describing what to expect at a genetic counseling session, and obtaining credible, current information about genetics were part of their current practice. Finally, involvement in emerging genomics practices such as pharmacogenetics (28.0%), direct-to-consumer genetic tests (discussing risks/benefits/limitations 16.7%, interpretation 14.1%), and whole genome sequencing (7.6%) was even more limited.

### Confidence in GM Skills

Self-reported confidence for these same GM skills was generally low (**Table 2**). Even for high involvement skills, confidence was moderate (ranging from 21.3% to 55.3%), while fewer than 5% agreed/strongly agreed they were confident in the emerging genomic practices listed above.

### Attitudes Toward GM

More than half (203/342, 59.4%) agreed/strongly agreed that they expected advances in GM to improve patients’ health outcomes and that they needed to keep up to date with advances in GM (179/343, 52.2%) and 43.1% (148/343) agreed it was important to learn about personalized patient care based on targeted or whole genome sequencing (**Table 3**). Fewer than half (124/342, 36.3%) agreed it was their responsibility to incorporate GM into practice or saw sufficient benefits to warrant testing for inherited adult onset disease (140/342, 40.9%). Only 15.2% (52/341) agreed or strongly agreed that genomics is an exciting part of practice. However, the majority agreed/strongly agreed that GM is going to make important contributions to the diagnosis and management of prenatal (269/342, 78.7%), pediatric (259/342, 75.7%), and adult onset conditions (215/341, 63.0%).

**Table 3:** Attitudes toward genomic medicine.

Statement	Agreed/Strongly agreed	
	N	%
Advances in genomic medicine will improve my patients’ health outcomes	203/342	59.4
I need to keep up to date with advances in genomic medicine	179/343	52.2
Important for me to learn about personalized patient care based on targeted or whole genome sequencing	148/343	43.1
As a primary care provider, it is my responsibility to incorporate genomic medicine into my practice	124/342	36.3
There are sufficient benefits to warrant testing for inherited adult onset diseases	140/342	40.9
I find genetics and genomics an exciting part of my practice	52/341	15.2
Genomic medicine is going to make important contributions to diagnosis and management of: Prenatal conditions Pediatric conditions Adult onset conditions	269/342 259/342 215/341	78.7 75.7 63.0

### Awareness of Genetic Services

Very few agreed/strongly agreed that they could identify useful sources of information regarding genetics for their practice (78/349, 22.3%) or could find information about genetic tests available within the health care system (74/348, 21.3%) (**Table 4**). The majority however, knew where to refer for various genetic disorders (prenatal 240/255, 94.1%; newborn screening 173/216, 80.1%; pediatric 241/294, 82.0%; adult onset 247/328, 75.3%), with most having referred for prenatal genetic issues or adult onset genetic disorders (prenatal 177/253, 70.0%; newborn screening 69/210, 32.9%; pediatric 106/282, 37.6%; adult onset 236/327, 72.2%).

**Table 4:** Awareness of genetic services.

Statement	Agreed/Strongly agreed	
	N	%
I can identify useful sources of information regarding genetics for my practice	78/349	22.3
I can find information about genetic tests available within healthcare system	74/348	21.3
	Yes*	
	**N**	**%**
Know where to refer patients for these disorders: Prenatal genetic disorders Newborn screening disorders Pediatric genetic disorders Adult onset genetic disorders	240/255 173/216 241/294 247/328	94.1 80.1 82.0 75.3
Have referred a patient to a genetics clinic for a personal or family history of any of these disorders: Prenatal genetic disorders Newborn screening disorders Pediatric genetic disorders Adult onset genetic disorders	177/253 69/210 106/282 236/327	70.0 32.9 37.6 72.2

*Includes only respondents who provide care in specified areas.

### Knowledge Regarding Clinical Genetic Disorders

The median knowledge score on the 10 clinical vignettes was 6/10 with a range from 0 to 10 (**Table 5**). On average, 31.0% indicated they were unsure of the answer.

**Table 5:** Clinical vignettes/knowledge questions regarding clinical genetic disorders.

Vignette (correct response is bolded)	Correct response	
	N	%
1. Suppose you had a patient whose aunt or grandmother on her father’s side carried the *BRCA1* gene mutation for breast/ovarian cancer syndrome. In your opinion, could your patient also be a carrier of this mutation? **a.** **Yes** b. No c. Not sure	181/339	53.4
2. In your opinion, what percentage of breast cancer patients has a *BRCA1* or *BRCA2* gene mutation? **a.** **< 10%** b. 10-50% c. 51-100% d. Not sure	206/339	60.8
3. In your opinion, what percentage of patients who carry a gene for hereditary non-polyposis colorectal cancer will actually go on to develop colorectal cancer? a. < 50% **b.** **≥50%** c. Not sure	153/338	45.3
4. A father and his son have the same inherited single gene disorder. The least likely mode of inheritance for this disorder is: **a.** **X-linked** b. Autosomal dominant c. Autosomal recessive d. Not sure	157/338	46.4
5. All of the following are absolute indications to offer a prenatal patient referral for genetic counseling EXCEPT: a. One parent is a carrier of a balanced chromosomal rearrangement b. Parental consanguinity **c.** **History of one prior pregnancy ending in miscarriage.** d. Family history of cystic fibrosis e. Not sure	276/337	81.9
6. The Society of Obstetricians and Gynaecologists of Canada recommends offering pre-conception or prenatal genetic screening for which disorder(s) to couples where only one member is of Ashkenazi Jewish descent? a. Tay-Sachs disease b. Canavan disease c. Familial dysautonomia **d.** **All of the above** e. Not sure	139/338	41.1
7. A young boy has behavioral problems and developmental delay. Which is the least likely genetic diagnosis? a. Williams syndrome b. Down syndrome c. Fragile X syndrome **d.** **Turner syndrome** e. Not sure	194/338	57.4
8. You’ve been monitoring a patient for a strong maternal history of colon cancer. During a routine gynecological exam, she corrects a note in her chart that a maternal aunt actually had endometrial cancer and not cervical cancer. This raises your index of suspicion to recommend genetic counseling for which hereditary colon cancer syndrome?	115/350	32.9
a. Familial juvenile polyposis b. Familial colitis **c.** **HNPCC (hereditary non-polyposis colon cancer) or Lynch syndrome** d. FAP (familial adenomatous polyposis) e. Not sure		
9. A 29-year-old female patient informs you that her husband is her maternal first cousin. She is concerned about the risks to their future offspring. You counsel her that: a. The chance for this couple to have a child with a congenital anomaly is about the same as population risk (2-3%) b. **The chance for this couple to have child with a congenital anomaly is about double the population risk (4-6%)** c. The chance for this couple to have a child with a congenital anomaly is significantly higher than the population risk (> 10%) d. Not sure	103/351	29.3
10. Please indicate which one of the following scenarios would be appropriate for referral to genetics: a. A patient’s family history is significant for dementia in her mother. The age of onset is 72 b. A patient reports a family history of dementia in her maternal grandfather in his early eighties and in her maternal aunt at age 67 c**.** **A patient reports a family history of dementia in her paternal grandfather in his sixties and in her paternal uncle in his fifties. Her father is age 48 and in good health** d. Not sure	250/347	72.0

### Genetics Resources

Resources “usually used” for information about genetics included Up to Date^®^ or similar internet sources, Google or Wikipedia (**Table 6**). Fewer than half used their local genetics clinic or local specialists. Resources that respondents indicated would be useful included local genetics clinic contact information (308/347, 88.8%), genetic referral (293/343, 85.4%), and testing (296/344, 86.0%) guidelines, information summaries for patients about genetic disorders (246/344, 71.5%) and disease-specific risk assessment tools (279/343, 81.3%). Over half (193/342, 56.4%) thought a genetics education website would be useful (results not shown). Respondents indicated their level of interest in a menu of education topics in GM listed in **Table 7**. More than half (205/355, 57.7%) expressed moderate to high interest in learning about new advances in genomic technologies.

**Table 6:** Resources usually used for information about genetics^*^.

Resource	N	%
Up to Date or similar internet source	183/346	52.9
My local genetics clinic/genetic counselor/geneticist	166/346	48.0
Internet search engine (e.g., Google)	159/346	46.0
Local specialists	114/343	33.2
Wikipedia	72/346	20.8
Local genetics clinic website	50/346	14.5
Genetests website	14/346	4.0

*Includes all respondents including FPs in focused practice.

**Table 7:** Genomics topics of interest to family physicians^*^.

Topic	Respondents reporting moderate or high interest	
	N	%
Genomic risk factors for common complex diseases (e.g. cancer, heart disease, diabetes	272/355	76.6
Genetics services in your area	267/353	75.6
Genetics of common single gene disorders (e.g. cystic fibrosis, hereditary breast and ovarian cancer)	266/356	74.7
Genetic testing (e.g. clinical utility, availability, how to order, benefits/harms, accuracy, interpretation)	255/355	71.8
Family history (e.g. taking a multigenerational history, red flags, assessing risk, recognizing patterns of inheritance)	249/356	69.9
Basic genetic concepts (e.g. inheritance, genes, mutation, penetrance, predisposition versus diagnosis)	219/356	61.5
New advances in genomic technologies entering clinical practice (e.g. “Direct-to-Consumer” genomic testing, whole genome sequencing, microarray)	205/355	57.7

*Includes all respondents including FPs in focused practice.

Contact with a local genetic counselor by telephone/fax or email (225/339, 66.4%) or a buddy system with a geneticist being available for questions (172/339, 50.7%) were the most popular suggestions for how to integrate GM into primary care practice. Less than half wanted a visiting genetic counselor providing educational sessions (118/339, 34.8%), a FP in their clinic with a special interest in genetics (73/339, 21.5%), or a genetic counselor in the clinic seeing patients (65/339, 19.2%) (results not shown).

There was a weak positive correlation between high knowledge and high confidence (Pearson correlation coefficient r = 0.227, p < 0.001). No demographic variables were associated with high confidence. Being age 50 or under (40.7% ≤50 vs 21.5% > 50, p < 0.001), female (38.2% vs 23.2% male, p = 0.005), in group practice (35.2% group vs 14.3% solo, p = 0.001), involved in teaching (36.7% teaching vs 21.7% not, p = 0.005), using an EMR (34.4% using EMR vs 16.0% not p = 0.002), having some formal genetics education (41.4% education vs 26.0% not, p = 0.009), and indicating interest in genetics (42.9% interest vs 27.7% not indicating interest, p = 0.036) were significantly associated with higher knowledge. Respondents who were involved in teaching (43.4% vs 28.1% not in teaching, p = 0.004), indicated interest in genetics (50.0% vs 33.6% not interested, p = 0.024), or had high confidence in the GM skills specified (50.9% vs 30.2% low confidence, p = 0.004), were more likely to agree/strongly agree that it was their responsibility to incorporate GM into their practices.

**Table 8** indicates predictors of high reported confidence in various clinical skills in GM. Participants who indicated they had an interest in genetics were twice as likely to have a high confidence score (≥5/10) (OR 2.17 95% CI 1.00–4.70, p = 0.05). Individuals who indicated an interest in genetics were also more likely to agree or strongly agree that advances in GM will improve patients’ health outcomes (OR 3.18, 95% CI 1.50–6.71, p = 0.002) and that it is their responsibility to incorporate GM into practice (OR 1.93, 95% CI 1.03–3.63, p = 0.042). (**Table 8**) Female FPs (OR 1.90, 95% CI 1.05–3.41, p = 0.033) and those indicating an interest in genetics (OR 2.01, 95% CI 1.01–3.98, p = 0.046) were also significantly more likely to have a high knowledge score (≥7/10) (**Table 8**).

**Table 8:** Confidence, attitudes, awareness, and knowledge regarding genomic medicine: significant results from binary logistic regression analysis.

Outcome variable	Covariate	Odds ratio	Lower 95% CI	Upper 95% CI	p-value
**Confidence (high: level 4 or 5)**					
Eliciting information about genetic conditions as part of family history	Female CE last 5 yrs	1.83 2.44	1.09 1.24	3.07 4.80	0.022 0.010
Identifying individuals with a genetic condition	Interest in genetics	2.35	1.21	4.58	0.012
Deciding who to offer genetics referral	Focused practice	0.38	0.17	0.88	0.024
Knowing where to refer for genetic assessment	Female Teaching CE last 5 yrs	1.69 1.69 2.36	1.01 1.01 1.17	2.84 2.83 4.73	0.048 0.046 0.016
Providing genetics education to patients	Age ≤50 Female Teaching	2.42 0.48 2.66	1.02 0.24 1.22	5.75 0.99 5.80	0.046 0.047 0.014
Providing support to patients with a genetic test result	Focused Practice CE last 5 yrs	0.34 3.14	0.14 1.59	0.82 6.21	0.016 0.001
Discussing benefits/risks of genetic testing with patients	CE last 5 yrs	2.47	1.09	5.57	0.030
Obtaining credible/current info about genetics	CE last 5 yrs	3.00	1.06	8.48	0.038
High confidence score (≥5/10)	Focused practice Interest in genetics	0.29 2.17	0.09 1.00	0.89 4.70	0.030 0.050
**Attitudes (agree or strongly agree)**					
Advances in genomic medicine will improve health outcomes	Female Interest in genetics	0.57 3.18	0.33 1.50	0.97 6.71	0.039 0.002
Need to keep up to date with advances in genomic medicine	Interest in genetics	3.23	1.63	6.37	0.001
Important to learn about personalized patient care based on whole genome sequencing	Female Use EMR Interest in genetics	0.56 2.06 3.50	0.33 1.06 1.80	0.94 3.99 6.81	0.029 0.033 <0.001
My responsibility to incorporate genomic medicine into practice	Interest in genetics	1.93	1.03	3.63	0.042
Genetics is an exciting part of my practice	CE last 5 yrs Interest in genetics	2.32 4.85	1.00 2.32	5.38 10.15	0.049 <0.001
**Awareness (agree or strongly agree)**					
Can identify useful sources of information	Genetics Education Interest in genetics	2.44 1.99	1.28 1.01	4.65 3.93	0.007 0.048
I know how to contact my local genetics centre	CE last 5 yrs	2.17	1.05	4.48	0.036
**Knowledge**					
High knowledge score (≥7/10)	Female Interest in genetics	1.90 2.01	1.05 1.01	3.41 3.98	0.033 0.046

CE, continuing education in genetics in last 5 years. Genetics education, some formal education in genetics.

CI, confidence interval; EMR, electronic medical record.

Those who indicated an interest in genetics were significantly more likely to indicate moderate or high interest in almost every type of education offered (**Table 9**). Those who use an EMR were more likely to find various guidelines, apps, and tools useful (**Table 9**). We compared demographic variables of those who indicated a special interest in genetics with those who did not. The only significant difference was that 32% of those with a special interest in genetics indicated they had a genetic condition in the family compared with 18% of those with no special interest (p = 0.15).

**Table 9:** Genomic medicine education and resources: significant results from binary logistic regression analysis.

Outcome variable	Covariate	Odds ratio	Lower 95% CI	Upper 95% CI	p-value
**Education (method of learning about genetics: moderate or high interest)**					
In person seminar, workshop, lecture	CE last 5 yrs Interest in genetics	0.46 2.60	0.22 1.10	0.94 6.18	0.033 0.030
Video conferencing of seminar, workshop, lecture	Teaching Interest in genetics	1.92 2.33	1.00 1.19	3.66 4.58	0.049 0.014
Didactic lecture on website	Interest in genetics	2.08	1.09	3.99	0.027
Podcast	Age ≤40	3.19	1.34	7.59	0.009
Problem-based small group learning modules	Urban Interest in genetics Condition in family	0.58 3.86 2.25	0.34 1.88 1.18	0.97 7.93 4.30	0.038 <0.001 0.014
Interdisciplinary learning environment	Age ≤40 Interest in genetics	0.43 2.13	0.21 1.14	0.90 3.99	0.024 0.018
Short observership with genetic counselor	Genetics education Interest in genetics	0.43 3.47	0.19 1.70	0.95 7.09	0.037 0.001
Genetics education sessions at practice	Interest in genetics	2.18	1.15	4.13	0.017
Genetics education website	Teaching Interest in genetics	0.51 2.13	0.30 1.08	0.89 4.20	0.018 0.030
**Genetics resources (useful or very useful for your practice)**					
Information summaries	Female	2.04	1.14	3.67	0.017
Downloadable MP3 audioclips/lectures/podcasts	CE last 5 yrs	0.35	0.14	0.90	0.029
CD ROMs	Age ≤40 CE last 5 yrs	0.28 0.31	0.12 0.11	0.70 0.86	0.006 0.025
Genetic testing guidelines	Use EMR	2.61	1.13	6.04	0.025
Disease specific risk assessment tools	Use EMR	2.14	1.00	4.59	0.050
EMR	Use EMR	6.32	3.18	12.57	< 0.001
Apps for smartphones and tablets	Use EMR	2.80	1.44	5.45	0.002
Web Widgets	Age ≤50	3.17	1.40	7.18	0.006
Genetics education website	Focused practice Interest in genetics	2.82 2.22	1.33 1.12	5.97 4.39	0.007 0.022

CE: continuing education in genetics. Table 9. Genomic Medicine Education and Resources: Significant Results from Binary Logistic Regression Analysis

## Discussion

This study offers a comprehensive view of FPs’involvement, confidence, attitudes, and resources needed in GM. The vast majority of participating FPs reported that key tasks in the delivery of traditional GM (eliciting family history, identifying patients with a genetic condition, deciding who should be offered genetic referral, knowing where to refer) were part of their current practice. The concern is that their confidence in these tasks was low. Fewer than half were confident in eliciting FH and knowing who to refer. There was a weak positive correlation between knowledge and confidence. Those who indicated they had continuing education in genetics in the past 5 years had significantly increased confidence in a number of GM skills. This lack of confidence has been shown in many studies spanning almost two decades (Suchard et al., 1999; Greendale and Pyeritz, 2001; Burke, 2004; McCahon et al., 2009; Carroll et al., 2011; Mainous et al., 2013; Rinke et al., 2014; Chambers et al., 2015) Fewer than 2/3 of participants in our study reported that evaluating or discussing genetic tests was part of their current practice. This is similar to a recent US study of PCPs where only 19% had ordered genetic testing, and 18% had consulted with a genetic counselor in the past 6 months, most frequently for cancer risk testing and prenatal testing. (Chambers et al., 2015) Many genetic tests are already in the primary care domain and with new advances in GM, it is likely more will be available to PCPs. It is also likely that limited genetics resources (e.g. genetics clinics with long wait times), and few genetic specialists and counselors, will push more genetic testing into PC practice and that genetics specialists will be looking to their PCP colleagues to take a bigger role in pre-test counseling and assessment.

Attitudes regarding GM were mixed. Over half the respondents agreed that GM is going to make important contributions to diagnosis and management and will improve health outcomes. However fewer than half (41%) of the responding FPs agreed there are sufficient benefits to warrant testing for inherited adult onset diseases, and were even less convinced that it was their responsibility to incorporate genomics into practice (26%). The literature is mixed in this regard with some reporting cautiously optimistic attitudes about genetic testing, citing its value for risk stratification, and that testing is likely to have impact on clinical practice in the future, (Mainous et al., 2013; Manolio et al., 2013; Chambers et al., 2015) and others expressing caution about the role of FPs in clinical genetics (Mathers et al., 2010) and wanting more evidence of clinical utility (Mainous et al., 2013). It is interesting that an interest in genetics was predictive of “positive” attitudes to GM, needing to keep up to date and incorporate GM into practice.

Our findings regarding some of the newer areas of GM are similar to those found in the literature. Not surprisingly, emerging areas such as pharmacogenetics, direct-to-consumer genetic testing, and whole genome sequencing were less likely to be part of current practice and confidence in these areas was low. Haga’s study of PCPs showed that most (73%) had heard of pharmacogenomics and anticipated its value in informing drug response (65%) (Haga et al., 2012), however only 13% felt well-informed and 67% were uncomfortable ordering a pharmacogenetic test. This study concluded that “primary care practitioners envision a major role for themselves in the delivery of pharmacogenomic testing but recognize their lack of adequate knowledge and experience about these tests,” (Haga et al., 2012) very similar to how providers see GM generally. A similar situation exists for direct-to-consumer genetic testing. Health care providers report low awareness and experience of direct-to-consumer genetic testing (Bernhardt et al., 2012; Ram et al., 2012; Goldsmith et al., 2013; Carroll et al., 2016a; Carroll et al., 2016b), however, many believe it will be helpful in patient management (Bernhardt et al., 2012; Powell et al., 2012a; Powell et al., 2012b). In Powell’s survey of PCPs, of 39% who were aware of direct-to-consumer genetic testing, 43% thought it was clinically useful. The majority (85%) were unprepared to answer patient questions and 74% wanted to learn more. (Powell et al., 2012a; Powell et al., 2012b) This is in contrast to a study of academic FPs who were concerned that direct-to-consumer genetic tests might cause more harm than benefit. (Mainous et al., 2013) Many patients however, plan to share their personalized genomic test results with their PCP (Van der Wouden et al., 2016) and report satisfaction with that encounter if they perceive that the PCP understands genetics and is willing to discuss test results. (Van der Wouden et al., 2016)

Addressing system issues has been highlighted as important to successful integration of genomics into primary care practice. (Mathers et al., 2010; Manolio et al., 2013; David et al., 2015) Less than a quarter of participating FPs indicated they could find information about genetics and available genetic testing, although encouragingly, most knew where to refer for genetic disorders. Fewer than half contacted their local genetics clinic for information, the majority used various internet resources. These findings speak to the challenge of educational initiatives, the need to enable providers to assess when genomic testing offers added value and will change patient outcomes (Manolio et al., 2013; David et al., 2015), and the need to strengthen the relationships between genetic centers and the PC community in order to make GM services more accessible.

Increasing skills and confidence in taking a FH should be a key priority for medical education at all levels. Family history is still relevant in the genomic era as it is key to risk assessment, informing appropriate screening, and identifying those who may benefit from genetics consultation. (Skirton et al., 2010; Doerr and Teng, 2012; Pyeritz, 2012; Korf et al., 2014) Opportunities should be sought to build on existing knowledge and skills in eliciting FH, to frame GM as part of ongoing skill development, not a specialized area of medicine dealing with “rare” diseases. (Botkin et al., 2015) Development of FH tools suitable for primary care, that are integrated into the EHR with clinical decision support, may facilitate this.

More efforts are needed to develop both effective education and practice strategies to enable PCPs to integrate GM into primary care. This needs assessment builds on existing literature to provide direction to educational initiatives. Core competencies in genetics for non-genetics health professionals have been proposed (Burke et al., 2009; Skirton et al., 2010; Houwink et al., 2013; Manolio et al., 2013; Korf et al., 2014) including taking a FH, risk assessment, when and how to order genetic tests, interpretation, pharmacogenetics, ethical dilemmas and psychosocial effects related to genetics, and insight into the organization and role of clinical genetics services (Houwink et al., 2011). Clearly the FPs in our study identified taking FH, knowing who to refer and supporting patients who received genetic results as their current role, suggesting that educational and practice strategies should focus in these areas. Our results would suggest that newer educational methods such as podcasts and web-based tools may be more appealing to younger physicians. There are limited studies of educational interventions in GM showing mixed effectiveness. (Rubanovich et al., 2018) They include studies of interactive web-based curricula and educational modules (Blazer et al., 2005; Blazer et al., 2011; Houwink et al., 2013; Bell et al., 2014; Houwink et al., 2014; Orlando et al., 2014; Reed et al., 2016; Paneque et al., 2017), FH and clinical support programs (Jackson et al., 2018), point-of-care tools and decision support (Carroll et al., 2011; Carroll et al., 2014), and push reflective e-learning (Carroll et al., 2016b). Several websites exist with genomics information and on-line educational programs for PCPs (GECKO www.geneticseducation.ca; Genetics in Primary Care Institute https://www.aap.org/en-us/advocacy-and-policy/aap-health-initiatives/Pages/Genetics-in-Primary-Care-Institute.aspx; Genomics Education Programme, www.genomicseducation.hee.nhs.uk; The Jackson Laboratory, https://www.jax.org/education-and-learning/clinical-and-continuing-education; Genetics/Genomics Competency Centre, www.g-2-c-2.org; Gen-Equip programme, www.primarycaregenetics.org). A recent systematic review of interventions providing genetics education for PCPs highlights some of the challenges in this area and the need for evaluation of educational initiatives to include changes in practice to see if they are effective in improving patient management. (Paneque et al., 2016) Generally, initiatives using effective continuing education strategies (interactive, case-based, skill focused, sequential reinforced learning) have been most successful. (Paneque et al., 2017)

The abundance of studies over the past decade demonstrating a continued lack of knowledge and confidence in GM among PCPs shows that education alone is not sufficient. As Feero says “Available studies suggest that development and maintenance of freely available high-quality genomics reference and educational materials is likely insufficient to ensure a meaningful increase in genomics competency among non-geneticist health providers.” (Feero et al., 2014) Among the cultural and infrastructure changes he recommends are efforts to address the usability of EHR to manage and interpret genomic information and the time/cost burden in practice. Burke has also addressed the slow introduction of personal genomics into practice. (Burke and Korngiebel, 2015) She describes several factors that contribute to “this translational gap between knowledge and clinical application” including an evidence deficit to support the use of some genetic tests, lack of clinical education and decision support for health care providers, and inflated expectations of the clinical benefit of GM, particularly in managing chronic complex diseases. She suggests using the principles of implementation science “which focuses on identifying and overcoming barriers associated with deploying and tailoring new interventions” as a means to address the gap between testing capability and practice, in those cases where evidence of utility is clear. (Burke and Korngiebel, 2015)

Our findings suggest that PCPs are open to changes in practice to facilitate GM. Over half our respondents thought that a telephone/fax/email helpline to a local genetic counselor or a “buddy system” where a designated geneticist was available to answer questions, would help them integrate GM into their practices. There is an emerging literature exploring how this might happen. (Battista et al., 2012; Houwink et al., 2013; Manolio et al., 2013; David et al., 2015) One such model used tailored genetics education outreach delivered by a genetic counselor to general practices over 1 year, including genetic update sessions, a responsive advice service, and referral guidelines. This service was evaluated positively by participants with continued utilization of the genetic counselor for advice following completion. (Drury et al., 2007) This type of model requires clinician acceptance and “reconfiguration of professional roles and responsibilities.” (Battista et al., 2012) Interestingly, the idea of a FP or nurse with a special interest in genetics in the clinic or a visiting genetic counselor to consult in the practice was less popular among our respondents. This may be due to the relative rarity of genetic conditions in primary care. Access to a genetics specialist has been positively associated with use of genetic testing for disease diagnosis or susceptibility, however many PCPs report they do not have access to genetics expertise. (Haga et al., 2013) It may be as Haga postulates that “access for some PCPs may be effectively limited if they are unfamiliar with these experts or have not had any clinical occasion to consult them.” Perhaps there is a role for counseling by phone, telemedicine or electronic consultation to enhance communication and contact. (Haga et al., 2013) As a result of this study, we developed a website containing evidence-based resources, including point-of-care tools, on GM for PCPs with clear information about how to access local genetic services (www.geneticseducation.ca). We are also exploring electronic consultation, questions directed to clinical geneticists by PCPs over a secure electronic platform, with response within 7–10 days, as a means for seeking clarification or guidance regarding clinical care in GM.

## Limitations

The main limitation to this study was the low response rate, bringing into question the generalizability of the results. Compared to the 2013 National Physician Survey in Canada (closest in time to the study), our study respondents were of similar age (median age 51 this study, 52 National Physician Survey), higher proportion female (47%/40%), slightly lower EMR use (74%/78%), and similar likelihood to be paid through an alternative funding arrangement rather than fee for service (49%/51%). (College of Family Physicians of Canada, 2013) This implies some similarity of our sample to Canadian FPs. Study respondents were very similar in age distribution to non-respondents. This study had more female respondents than non-respondents (respondents 47% female, non-respondents 40% female). The random sample of 100 non-respondents that we contacted in order to adjust our response rate was 39% female, similar to our overall non-responder rate. The age distribution of the sample of 100 non-respondents was similar to the overall non-respondents. The low response rate may have been due to the length of the survey, possibly suggesting that those with more interest or knowledge of GM completed the survey. If this is the case, our results raise even more questions regarding FPs’ assessment of the clinical value of genetic tests and their readiness to incorporate GM into busy primary care practices. This study was conducted in one province in Canada, so its generalizability to PCPs in other countries is unknown.

## Conclusions

This study shows that FPs see a role for themselves in taking FH, identifying individuals with a genetic condition, making appropriate referrals and supporting patients following genetic test results. They continue to lack the knowledge and confidence in GM skills needed for practice, particularly in the emerging areas of GM. They are somewhat optimistic about the contribution GM may make to patient care, but express caution about its current clinical benefits. Our study suggests that there is a need for more evidence of clinical utility of genetic tests, educational resources which can be integrated into primary care practice, clinical decision supports, and improved communication with genetic specialists. Resources need to include the basic skills for delivering GM (e.g. referral guidelines and testing criteria) as well as the advancing areas of pharmacogenetics, direct-to-consumer genetic testing, and whole genome sequencing.

## Data Availability Statement

The datasets generated for this study are available on request to the corresponding author.

## Ethics Statement

The studies involving human participants were reviewed and approved by Children’s Hospital of Eastern Ontario Research Ethics Board. Written informed consent for participation was not required for this study in accordance with the national legislation and the institutional requirements.

## Author Contributions

JC, JA, SM, FM, BW, JP, and DT substantially contributed to conception and design, analysis and interpretation of data, and drafting the article and gave final approval to the version to be published. SM and JP contributed to acquisition of data.

## Funding

This study was funded by Genetics Education Canada—Knowledge Organization (GEC-KO) which is supported by funding from the Children’s Hospital of Eastern Ontario (CHEO).

## Conflict of Interest

DT is a consultant for mdbriefcase.com and for the Center for Effective Practice. 

The remaining authors declare that the research was conducted in the absence of any commercial or financial relationships that could be construed as a potential conflict of interest.
